# Data-driven modelling approach to circadian temperature rhythm profiles in free-living conditions

**DOI:** 10.1038/s41598-021-94522-9

**Published:** 2021-07-22

**Authors:** Jari Lipsanen, Liisa Kuula, Marko Elovainio, Timo Partonen, Anu-Katriina Pesonen

**Affiliations:** 1 Sleepwell Research Program, Faculty of Medicine, University of Helsinki, Helsinki, Finland; 2 Department of Public Health and Welfare, Finnish Institute for Health and Welfare, Helsinki, Finland

**Keywords:** Computational biology and bioinformatics, Psychology

## Abstract

The individual variation in the circadian rhythms at the physiological level is not well understood. Albeit self-reported circadian preference profiles have been consolidated, their premises are grounded on human experience, not on physiology. We used data-driven, unsupervised time series modelling to characterize distinct profiles of the circadian rhythm measured from skin surface temperature in free-living conditions. We demonstrate the existence of three distinct clusters of individuals which differed in their circadian temperature profiles. The cluster with the highest temperature amplitude and the lowest midline estimating statistic of rhythm, or rhythm-adjusted mean, had the most regular and early-timed sleep–wake rhythm, and was the least probable for those with a concurrent delayed sleep phase, or eveningness chronotype. While the clusters associated with the observed sleep and circadian preference patterns, the entirely unsupervised modelling of physiological data provides a novel basis for modelling and understanding the human circadian functions in free-living conditions.

## Introduction

Recognizing individual differences in the functions of circadian clocks has become increasingly relevant, as their impact on individual health and well-being has become better understood^1^. New knowledge on circadian clocks has created potential for improving human health also in a wider context, especially through the translation of circadian timing concepts to medical practice^2^. However, investigation of individual circadian periodicity in free-living and ecologically valid conditions, where individuals decide on their sleep–wake rhythms in the presence of different time-givers of their natural environment, lags behind the growing need. While the self-sustained endogenous circadian rhythms have a periodicity of near-24 h, there are marked differences in circadian rhythm manifestations across individuals, which are likely to stem from environmental^3^, behavioural^4,5^ and genetic inputs^6^. However, there is clearly a shortage of knowledge of the dynamics and recognizable patterns of circadian rhythm variation in free-living, ecologically valid conditions^7^. This study addresses this challenge by applying data-driven mathematical modelling that is not restricted by existing conceptualizations of chronotype, circadian preferences or diagnoses^8^, but aims at finding novel features or circadian oscillation patterns that a priori undefined groups of individuals may have in common.

In free-living conditions, measuring temperature with a wireless thermologger attached to skin is a feasible assessment modality of the circadian rhythms, period, and sleep propensity^9^ in population studies where the golden standard assessment method from the core temperature cannot be used. Thus far, thermologger data have been associated with diurnal preference only weakly^10^. However, these data have not been previously used exploratively, and there is a shortage of knowledge on how the circadian body temperature rhythm coincides with the actualized sleep–wake rhythm, and whether distinct sleep–wake rhythm profiles exist as being based on the temperature profile. Until now, only one study has used circadian temperature trajectories to characterize distinct profiles of individuals. They found three different chronotype profiles based on the timing of the temperature rhythm, rest-activity cycles and body position among young children aged 8–12 years. The more evening-oriented children had lower amplitudes in temperature rhythms, lower melatonin values, later and more irregular sleep–wake schedules as well as increased metabolic risks with higher concentrations of insulin and glucose in saliva and higher serum concentrations of triglycerides and total cholesterol^11^. However, their study focused only on the acrophase of temperature rhythm for subsequent categorization into groups on the basis of the acrophase’s tertiles, which might be insufficient for mathematical modelling of highly dimensional time series of intraindividual temperature variation^12^.

The current study offers a novel, entirely data-driven approach to mathematically model ambulatory circadian temperature time series data in free-living, ecologically valid conditions. We explore with diverse multivariate time-series clustering methods and stochastic learning tools, whether there are reproducible clusters of individuals having a similar pattern of circadian temperature variation, and how these clusters are associated with the actualized sleep–wake patterns, self-reported circadian preference, and very late timing for sleep as indicated by having symptoms of delayed sleep phase (DSP).

## Results

### Optimal cluster solution of circadian temperature

As we assumed, there was variation in the number of temperature time series clusters suggested by different fit measures. Davies–Bouldin index suggested two clusters, whereas Dunn and Silhouette index suggested that optimal number of clusters would be three.

**Figure 1:**
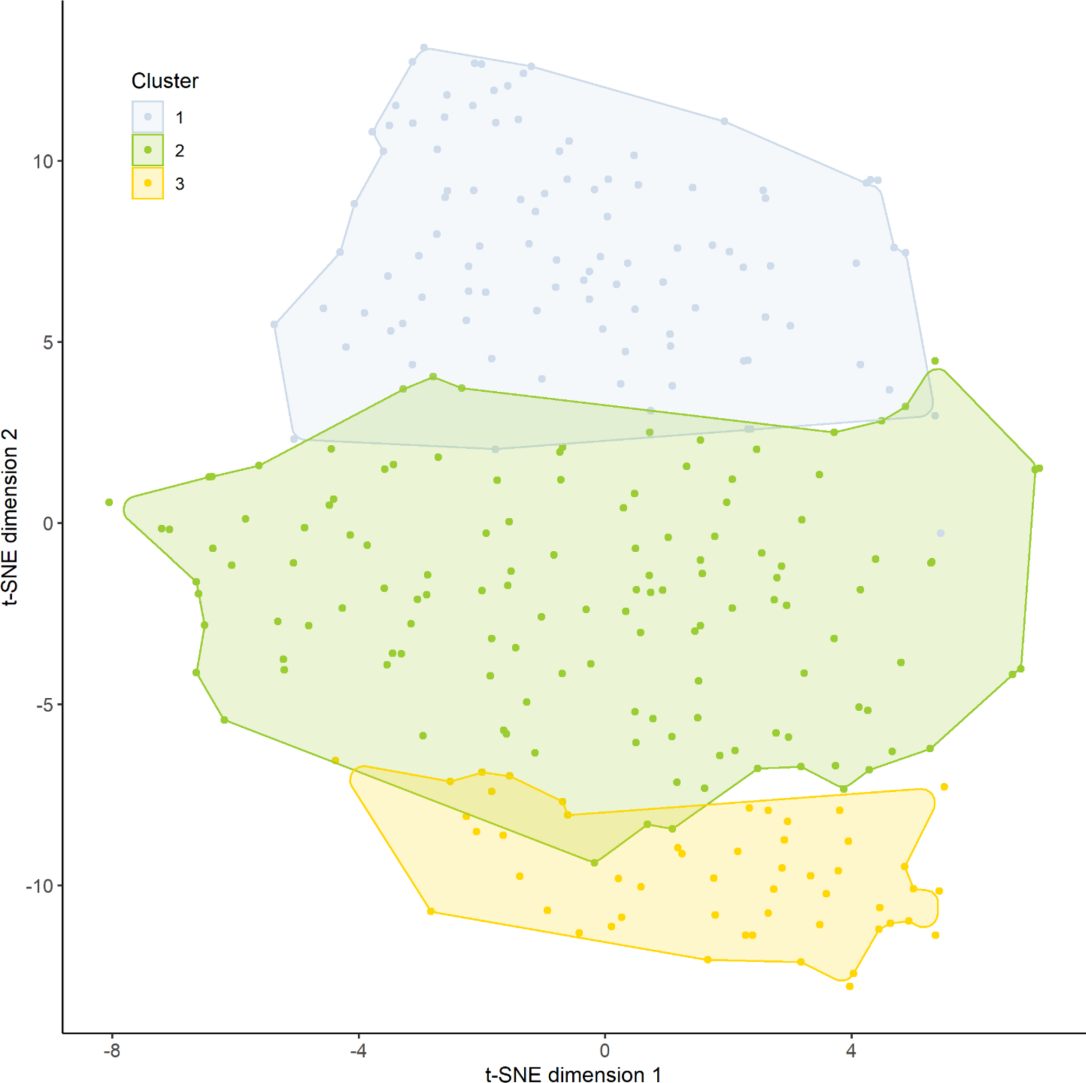
Comparison between t-SNE clustering and k-medoids clustering with three temperature time series clusters. Shaded area represents the convex hull of observations within a specific cluster. Figure produced using R Core Team (2021). R: A language and environment for statistical computing. R Foundation for Statistical Computing, Vienna, Austria. https://www.R-project.org/.

There was also variation when dimensionality of the data was inspected using principal components analysis based Euclidean distances between Fourier parameters of individual time series. Root mean squared error of approximation (RMSEA) was under acceptable level of 0.05 when number of clusters was three (RMSEA = 0.049), but empirical Bayesian information criteria achieved its minimum when the number of clusters was six. Due to this variation of the traditional fit measures, a number of clusters was also inspected using t-distributed stochastic neighbour embedding (t-SNE) to the original time series data, and this suggested three clusters (Fig. 1). Increasing the number of clusters resulted less distinct cluster borders^13^.

**Figure 2:**
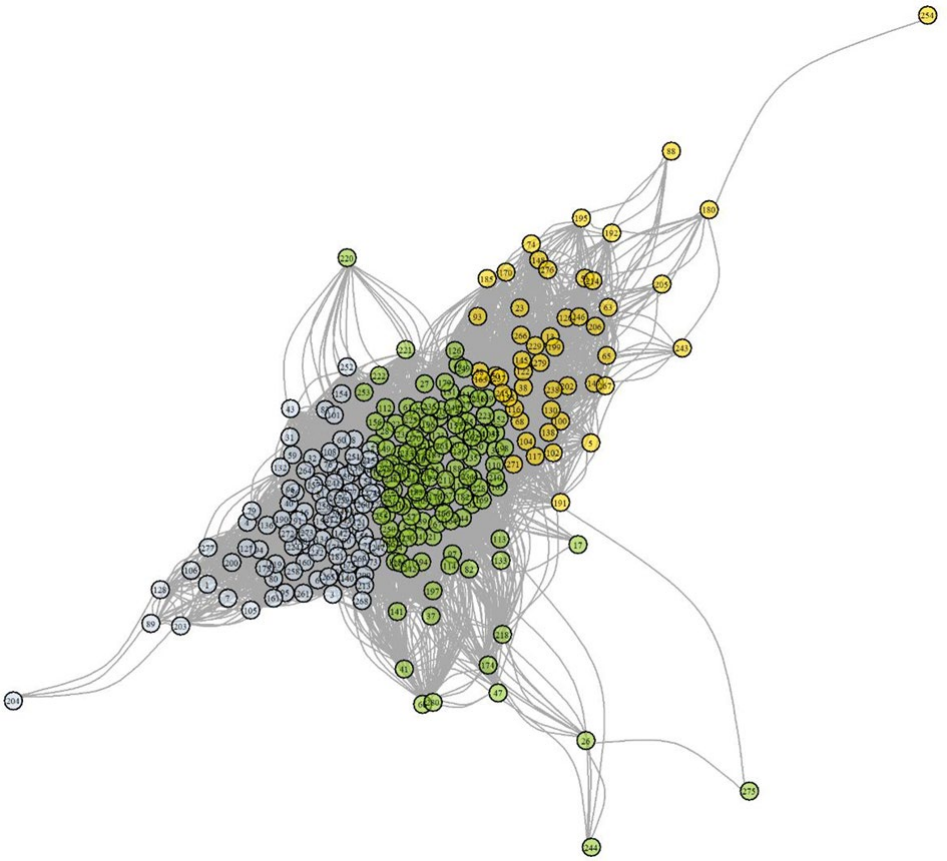
Network structure of temperature time series in the three-cluster solution. Network layout was determined using Fruchterman–Reingold algorithm. Gray, green, and gold colors represent clusters 1, 2, and 3, respectively. Figure produced using R Core Team (2021). R: A language and environment for statistical computing. R Foundation for Statistical Computing, Vienna, Austria. URL https://www.R-project.org/.

The three-cluster solution was also optimal based on graphical inspection of network analysis, which was estimated using Euclidean distances between Fourier parameters of individual time series. The three-cluster k-medoid solution, where network layout was determined using Fruchterman–Reingold algorithm^13^, showed clear clustering of network nodes (Fig. 2).

### Cluster characteristics and association to demographic characteristics

**Figure 3:**
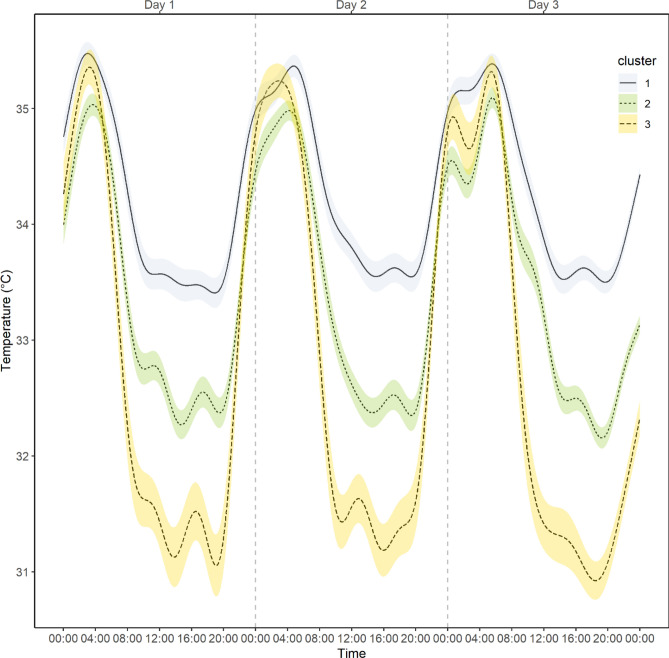
Cluster means and confidence intervals of the circadian temperature clusters based on original temperature measures. Confidence intervals: ± 1 standard error. Figure produced using R Core Team (2021). R: A language and environment for statistical computing. R Foundation for Statistical Computing, Vienna, Austria. https://www.R-project.org/.

The observed cluster sizes were 100, 130 and 51 participants in clusters 1–3, respectively. Table 1 presents the demographic characteristics of the participants in each cluster. There were no significant between cluster differences in age or body mass index (BMI), nor significant differences in sex (*p* > 0.05), although sex was close to statistically significant after taken account the false discovery rate correction, meaning that probability that member of Cluster 2 was a male (percentage of males: 36%, 95% confidence interval (CI) 28–48%) was higher compared to Cluster 3 (percentage of males: 17%, 95% CI 9–31%). The Time series of the cluster mean values and the 95% confidence intervals are presented in Fig. 3.

**Table 1:** Associations between clusters and demographic characteristics.

	Cluster	Mean/probability	95% lower CI	95% upper CI	*p *value	FDR corrected *p *value
Sex (male)	1	0.27	0.19	0.38	0.041	0.057
	2	0.36	0.28	0.45		
	3	0.17	0.09	0.31		
Age	1	16.86	16.74	16.98	0.326	0.36
	2	16.81	16.71	16.91		
	3	16.96	16.79	17.13		
BMI	1	22.04	21.29	22.78	0.508	0.533
	2	21.47	20.83	22.11		
	3	21.64	20.59	22.69		

Estimates are presented either as probabilities (sex), or as group averages (age and BMI). Confidence intervals are calculated using bias corrected bootstrapping with 1000 bootstrapped resamples. False discovery rate corrected *p *values take account all omnibus analysis presented in article.

*BMI*  body mass index (weight per square height, kg/m^2^), *CI* confidence interval, *FDR* false discovery rate.

### Comparison of the predictive power between the clusters and the Cosinor parameters

Clusters were characterized by examining cluster differences in estimated Cosinor parameters. All clusters differed significantly from each other in terms of Mesor (FDR corrected *p* < 0.001). Cluster 1 had the highest average temperature (M = 34.29, 95% bootstrap CI 34.22–34.35) compared to the average temperature of Cluster 2 (M = 33.46, 95% bootstrap CI 33.40–33.51) and Cluster 3 (M = 32.80, 95% bootstrap CI 32.71–32.89). All clusters also differed from each other in terms of Amplitude (FDR corrected *p* < 0.001). Cluster 3 had on average highest amplitude (M = 2.09, 95% bootstrap CI 1.96–2.21) compared to the cluster 2 (M = 1.38, 95% bootstrap CI 1.30–1.45) and Cluster 1 (M = 1.02, 95% bootstrap CI 0.93–1.10). There were no significant differences between the clusters in period, bathyphase or in acrophase (FDR corrected *p* > 0.10).

In addition, we compared association between the temperature clusters and the observed bathyphase (observed nadir time) and acrophase (observed peak time) on loess smoothed observed data (Table 2). Although Cluster 3 had latest bathysphere (M = 14:17, 95% bootstrapped CI 13:36–14:58) the difference was not statistically significant (FDR corrected *p* = 0.53) compared to Cluster 2 (M = 13:59, 95% bootstrapped CI 13:34–14:24) or Cluster 1 (M = 13:36, 95% bootstrapped CI 13:06–14:05). However, there were statistically significant differences in observed acrophase (FDR corrected *p* = 0.038). Although bootstrapped confidence intervals slightly overlapped, the observed acrophase of Cluster 1 (M = 02:40, 95% bootstrapped CI 02:14–03:06) and Cluster 2 (M = 02:34, 95% bootstrapped CI 02:12–02:56) was significantly different compared to Cluster 3 (M = 02:02, 95% bootstrapped CI 01:26–02:38) based on post hoc analysis.

**Table 2:** Associations between clusters and Cosinor model parameters.

	Cluster	Mean	95% lower CI	95% upper CI	*p *value	FDR corrected *p *value
Period (h)	1	25.75	25.15	26.35	0.679	1.00
	2	25.76	25.25	26.27		
	3	25.33	24.49	26.17		
Mesor (°C)	1	34.29	34.22	34.35	< 0.001	< 0.001
	2	33.46	33.40	33.51		
	3	32.80	32.71	32.89		
Amplitude	1	1.02	0.93	1.10	< 0.001	< 0.001
	2	1.38	1.30	1.45		
	3	2.09	1.96	2.21		
Bathyphase based on cosinor model (hh:mm)	1	14:52	14:27	15:16	0.101	0.149
	2	14:43	14:22	15:04		
	3	14:07	13:33	14:41		
Bathyphase based on smoothed data (hh:mm)	1	13:35	13:06	14:05	0.492	0.528
	2	13:59	13:34	14:24		
	3	14:17	13:36	14:58		
Acrophase based on cosinor model (hh:mm)	1	2:16:15	1:47:12	2:45:18	0.154	0.193
	2	1:50:36	1:25:43	2:15:29		
	3	1:28:44	0:47:53	2:09:36		
Acrophase based on smoothed data (hh:mm)	1	2:40:23	2:14:35	3:06:11	0.017	0.038
	2	2:34:30	2:12:24	2:56:37		
	3	2:02:20	1:26:02	2:38:37		

Bathyphase and acrophase are presented in time units. Both cosinor model estimates and observed times from loess smoothed data are presented. Confidence intervals calculated using bias corrected bootstrapping with 1000 bootstrapped resamples. False discovery rate corrected p-values take account all omnibus analysis presented in article.

*CI * confidence interval, *FDR* false discovery rate.

### Associations between the clusters, delayed sleep phase and self-reported circadian preference

Based on the logistic regression analysis, the association between the temperature clusters and a concurrent DSP was statistically significant (FDR corrected *p* < 0.001). Approximately 68% of members of Cluster 2 belonged to DSP group [95% Bootstrapped CI 59–75%] and based on post-hoc analysis the difference of DSP probability was significant compared to both cluster 1 where approximately 46% [95% Bootstrapped CI 36–56%] of cluster members had DSP and Cluster 3 where approximately 37% [95% Bootstrapped CI 24–52%] of cluster members had DSP.

**Figure 4:**
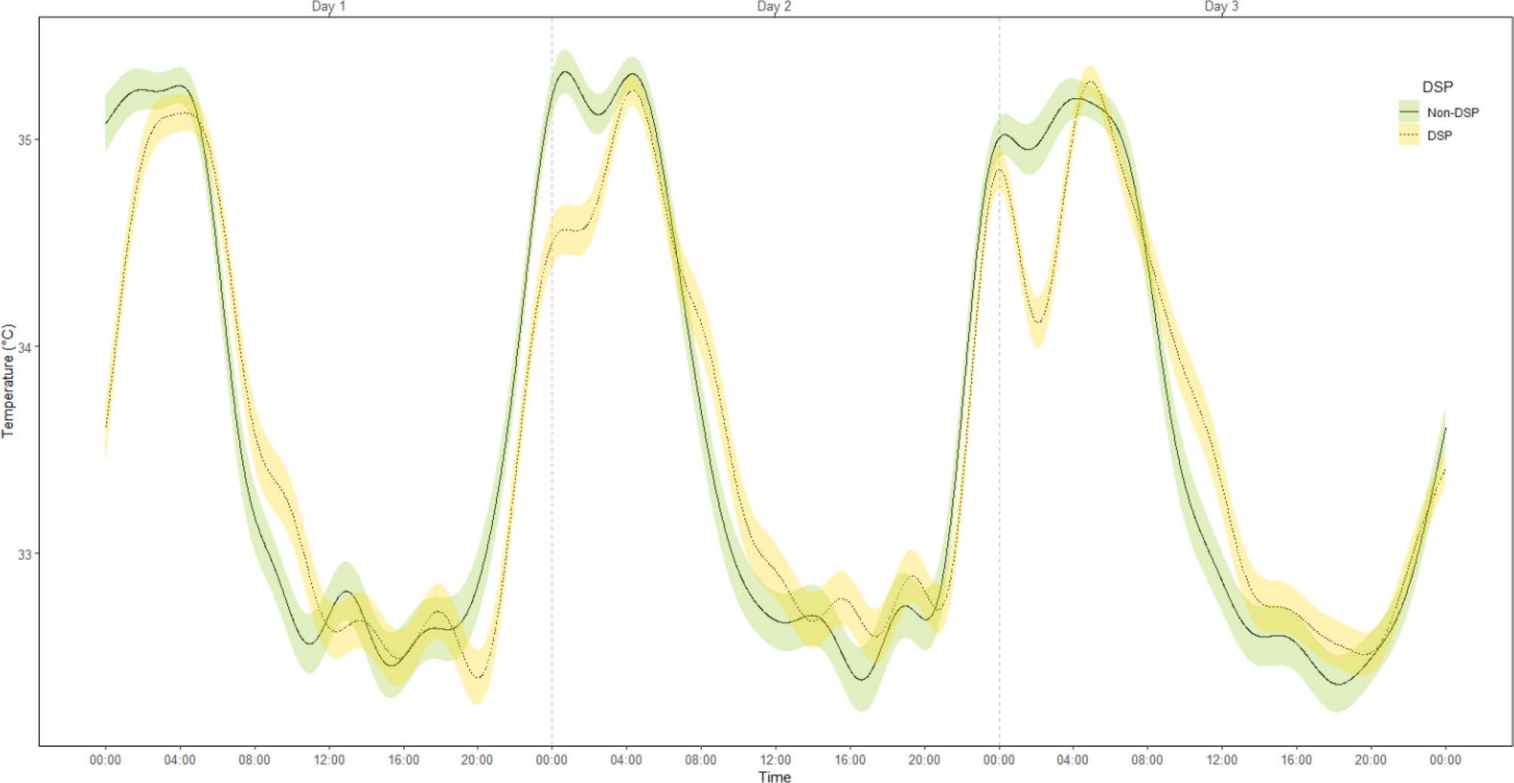
Means, and confidence intervals of original temperature measures in individuals with DSP characteristics and without. Confidence intervals: ± 1 Standard error. Figure produced using R Core Team (2021). R: A language and environment for statistical computing. R Foundation for Statistical Computing, Vienna, Austria. https://www.R-project.org/.

We also examined association between DSP and individual cosinor parameters, so that we could compare the predictive power between time series clustering and traditional individual cosinor analysis. Based on the results of logistic regression where all individual cosinor parameters were used as predictors of DSP, Mesor and amplitude were significant predictors of DSP, both having a negative relationship to DSP (OR_mesor_ = 0.48, 95% bootstrapped CI 0.28–0.78, FDR corrected *p* = 0.03; (OR_amplitude_ = 0.34, 95% bootstrapped CI 0.18–0.59, FDR corrected *p* < 0.01). No association in terms of period (OR = 1.04, 95% bootstrapped CI 0.85–1.16, FDR corrected *p* < 0.84) nor acrophase (OR = 1.04, 95% bootstrapped CI 0.89–1.21, FDR corrected *p* = 0.59) was found. The average observed bathyphase (time) for DSP group 14:05 (95% bootstrapped CI 13:39–14:29) and for non-DSP group 13:35 (95% bootstrapped CI 13:13–14:00) was not statistically significant. The average observed acrophase (time) was statistically significantly different in DSP group (mean = 03:18, 95% bootstrapped CI 02:59–03:39) compared to non-DSP group (mean = 02:05, 95% bootstrapped CI 1:46–2:25). The average period length for DSP group was 25 h 47 min (95% bootstrapped CI 25:19–26:27) and for non-DSP group 25 h 32 min (95% bootstrapped CI 25:18–25:46). Figure 4 displays the circadian profiles in DSP and non-DSP groups.

We also examined whether the clusters have any additional value compared to traditional individual cosinor parameters. To examine this question, we ran an additional model where we examined the association between the temperature clusters and DSP when all estimated cosinor parameters were controlled from the analysis. We found that the pairwise difference between Cluster 1 and 2 remained statistically significant (FDR corrected *p* = 0.015) but difference between Cluster 2 and 3 was no longer significant (FDR corrected *p* > 0.10). These results indicate that the sinusoid form of circadian rhythm is not the only affecting aspect when predicting DSP, although the relationship between the amplitude and DSP was also a significant predictor even when temperature clusters were included in the model [OR = 0.51, 95% bootstrapped CI 0.2–0.99, FDR corrected *p* = 0.045], indicating that the association between amplitude and DSP was negative.

In terms of the self-reported circadian preference, MEQ, there were statistically significant cluster differences in MEQ sum score (FDR corrected *p* = 0.036). Based on the results of the pairwise comparisons, Cluster 3 had highest average sum score, indicating a higher preference to morningness [mean = 14.38, 95% bootstrapped CI 13.27–15.48] compared to the Cluster 2 [mean = 12.43, 95% bootstrapped CI 11.76–13.10, 2 vs. 3 comparison FDR corrected *p* = 0.003] and compared to the Cluster 1 [M = 12.71, 95% bootstrapped CI 11.94–13.49, 1 vs. 3 comparison FDR corrected *p* = 0.016]. When cosinor parameters were controlled for, the difference between Clusters 2 and 3 remained significant [FDR corrected *p* = 0.011] and all associations between cosinor parameters were non-significant (*p* > 0.10), regardless of whether temperature cluster was included in the model or not.

### Associations between the clusters and the actualized sleep quantity and quality

Associations between clusters and individual sleep characteristics are presented in Table 3. Members of the Cluster 1 differed significantly from the members in Clusters 2 and 3 in sleep timing: they had earlier sleep onset, sleep midpoint, and sleep offset as well as the timing of their actualized sleep–wake rhythm over the 8-day measurement period was more regular. In terms of regularity: clusters 1 and 3 did not differ from each other significantly. There were no significant cluster differences in sleep quality measures.

**Table 3:** Associations between clusters and sleep characteristics.

	Cluster	Mean	95% lower CI	95% upper CI	*p *value	FDR corrected *p *value
**Sleep duration**						
Assumed sleep (hh:mm)	1	07:59	07:47	08:11	0.02	0.04
	2	07:37	07:26	07:47		
	3	07:42	07:25	07:58		
**Sleep quality**						
Wake after sleep onset (hh:mm)	1	00:52	00:47	00:57	0.233	0.271
	2	01:01	00:55	01:07		
	3	00:55	00:52	00:58		
Sleep onset latency (hh:mm)	1	00:18	00:13	00:25	0.115	0.145
	2	00:22	00:17	00:28		
	3	00:17	00:15	00:20		
**Timing**						
Sleep onset (hh:mm)	1	00:12	23:54	00:30	< 0.001	< 0.001
	2	02:03	01:41	02:22		
	3	00:48	00:36	01:00		
Sleep midpoint (hh:mm)	1	04:02	03:47	04:15	< 0.001	< 0.001
	2	06:02	05:41	06:23		
	3	04:40	04:31	05:50		
Sleep offset (hh:mm)	1	07:54	07:43	08:08	< 0.001	< 0.001
	2	10:06	09:40	10:32		
	3	08:35	08:26	08:44		
**Regularity**						
SD of sleep onset (min)	1	63.48	55.81	71.80	0.027	0.046
	2	83.99	72.32	96.50		
	3	70.86	65.29	76.67		
SD of sleep midpoint (min)	1	71.40	64.20	78.00	0.029	0.046
	2	80.40	74.40	85.80		
	3	67.20	58.20	76.80		

Confidence intervals are calculated using bias corrected bootstrapping with 1000 bootstrapped resamples. False discovery rate corrected *p *values take account all omnibus analysis presented in article.

## Discussion

Circadian rhythms influence our behaviour and status of health in profound ways. Maintaining a regular sleep–wake rhythm is clearly beneficial both in disease prevention^14–16^ and better disease management^8^. A synchronization of the activity and sleep periods according to the endogenous circadian rhythms optimize sleep^17^, cognitive processing^18^, metabolic functions^19^, immune functions^2^ and mood^20^. While individuals demonstrate variation in their subjective circadian preference and phase of entrainment, or chronotype^21^, similar variation at the level of biological markers of circadian rhythms is not well understood^22,23^. Assessment of the individual circadian periodicity in free-living and ecologically valid conditions, where individuals decide on their sleep–wake rhythms under the influence of different time-givers of their natural environment, lags behind the growing need. Here, we demonstrate with unsupervised network and cluster analytic methods individual profiles in circadian temperature rhythm, as well as investigate how this information is related to actualized sleep–wake rhythms, circadian preference and to a condition of subclinical delayed sleep phase disorder.

Our approach refines the endogenous circadian temperature analytics, considers the entire individual variation in circadian temperatures, and applies a bottom-up, explorative and data-derived approach. The data-driven approach to define distinct circadian profiles is particularly valuable, as it is not affected by any concurrent information on the individuals nor any predefined criteria based on existing theories^10,24–27^. It opens novel pathways to study and exploit circadian rhythmicity parameters in free-living conditions.

The unsupervised (bottom-up) nonlinear dimensionality reduction (clustering) technique is especially well-suited for embedding high-dimensional data. Associating the observed reduced cluster solution with a large variation of observed and self-reported sleep data provided concurrent validity indicators.

We demonstrate the existence of three distinct clusters of individuals, based on the mathematical modelling of their circadian temperature variation in their natural, free-living conditions. Instead of relying on one or two separate indicators, the current approach embeds the entire dynamics of the circadian time series. Cluster 3, for instance, is characterized by the greatest amplitude of circadian temperature, earliest acrophase and lowest mesor, and basing on the concurrent validity indicators, it associates with the most regular and earliest-timed sleep–wake pattern. While this cluster had also the shortest circadian period length, this aspect was not a central indicator in defining the cluster membership. The two other clusters (Cluster 1 and Cluster 2) were lower in amplitude, higher in mesor, and clearly less optimal in terms of the actualized sleep–wake rhythms. The Cluster 1 had the lowest amplitude and highest mesor, whereas Cluster 2 situated between Clusters 1 and 3 in these respects. The observed timing of the acrophase or oscillation peak differed across the clusters, such that Clusters 1 and 2 were later timed in comparison to the Cluster 3.

Prior reports on the circadian temperature rhythm from populations with DSP are very few, and they rely mostly on very few clinically validated disorders (DSP disorder, DSPD). A common observation in these studies is a longer circadian period in DSPD compared to healthy controls. One of the first studies showed that chronotherapy in individuals with a DSPD (N = 4) shortened the circadian period from 25.7 to 24 h^24^. Another case study^25^ measuring free-running rhythm for 17 days found that the patient with a DSPD had a circadian period length almost an hour longer (25.4 h) than in 3 healthy controls (24.4 h), and that the phase angle between minimum core temperature and the sleep phase was clearly deviant. The studies by Micic et al*.*^26,28^ also found 15 min longer circadian periods in DSPD compared to controls in both their studies performed under a modified constant routine of approximately three days. One study^27^ in adults associated a later chronotype with later timing of the bathyphase, whereas another study^10^ found only a modest association between eveningness and higher overall temperature.

The current study indicated a non-significant difference of 15 min in the circadian period between those with and without concurrent DSP, a difference being similar to the one reported by Micic et al*.* in their clinical study^26^. While all three observed clusters included individuals with DSP characteristics, the likelihood was greatest to belong into Cluster 2 (with 67% having DSP), which had an average amplitude and mesor, and latest timed acrophase. A visual inspection within the clusters showed that individuals with DSP characteristics had somewhat lower amplitudes in Cluster 3, and longer period lengths in Clusters 2 and 1. Cluster 3 was the most optimal in terms of concurrent sleep behaviour, and most uncommon for the DSP characteristics. While Cluster 2 was closer to Cluster 1 than Cluster 3 in many aspects, it was the latest timed and, indeed the most common for DSP.

The current study differs methodologically from studies on circadian rhythm that apply only a cosinor analysis to model circadian rhythmicity^11^. The most popular cosinor analysis is a parametric method; even a simple visual inspection of observed data or even of smoothed data, reveals that in many subjects, the circadian variation of temperature does not follow the traditional cosinor model. While other more complex parametric methods exist, e.g., one can estimate both within-day and between-day rhythms within multi-component cosinor framework^29^, the limitations of parametric models still persist. Also nonparametric approaches are available, but most of them aggregate data across all available days and not day-by-day^30^.

We propose a more direct method. Instead of trying to fit in an a priori theoretical model and assume that all discrepancies between the model and the observed data are simply due to measurement error, an improved approach utilizes clustering methods directly from observed data to identify subjects with similar circadian patterns. We observed that the circadian clusters were associated with the criterion outcomes (DSP and MEQ) even after adjusting for individual cosinor parameters. This supports the argument that not all the essential variation in circadian rhythmicity is reducible to a single component cosinor model. At the same time, it would be useful to have even more concurrent, criterion and predictive validity indicators beyond the used sleep measures. In addition, we used the traditional cosinor parameters to interpret specific cluster features, but witnessed their limitations for example in estimating acrophase: there was an association between the clusters and the observed acrophase, but not with a cosinor-derived acrophase.

We acknowledge some limitations in our study. Our definition of DSP is not based on clinical evaluation and lacks a longitudinal assessment of its stability. In addition, including measurements of dim light melatonin onset would have brought an additional aspect to the study of concurrent validity in terms of having a golden standard comparison method. Our study also lacks measurement of the light exposure, which would have been a valuable variable to include. Light exposure, as well as individual differences in sensitivity to it, is known to influence circadian rhythms and their disturbance^31–33^. Our sample of participants is homogenous in age, which can be an asset in finding valid and meaningful interindividual differences, but it may reduce external validity as this age group is influenced by circadian rhythm regulation problems more than other specific age groups, or the general population. Also having more females than males in the sample may influence the results. We also acknowledge that temperature rhythms are profoundly influenced by movement, sleep–wake behaviour, the environment, and various internal factors such as stress. The crucial question how these various factors interact with the circadian biology cannot be evaluated in this kind of study design. The concept of circadian period thus refers here to a broader concept of periodicity than assessed in a controlled laboratory study. Finally, directionality between the sleep–wake and temperature profiles remain uncertain.

## Conclusions

The concepts of circadian preference and chronotype have profoundly influenced our way of thinking human behaviour as being affected by individual characteristics of circadian periodicity. However, these concepts in relation to physiological parameters have only been studied to a modest degree^34–37^. Most importantly, the concept of circadian (or diurnal) preference to timing of the daily activities is grounded on human experience, not on a physiological assessment, and the concept of chronotype (phase of entrainment) is based on the half time of the primary sleep period, being subject to a conscious decision on sleep schedules. Here, we demonstrate an alternative, data-driven approach to find distinct profiles of circadian rhythmicity in free-living conditions. We identify three distinct patterns of circadian oscillations, of which the one having the greatest amplitude, the lowest mesor, and the earliest timing was associated with the most adaptive sleep–wake patterns. The association of the oscillation profile with the actual sleep–wake rhythm was not linear, such that the lowest amplitude cluster would be the worst sleepers. The most delayed sleep–wake rhythms were most common in Cluster 2, characterized by an average amplitude, a high mesor, and a late-timed acrophase of temperature. The entirely unsupervised modelling of physiological data provides clearly a new basis for understanding human circadian functions. This contribution can profoundly influence the way circadian periodicity is exploited in future studies as well as pave the way for how this information will be applied in medical and pharmaceutical care.

## Methods

### Participants

The research sample represent a subsample of the population-based cohort study SleepHelsinki, that used the Finnish Population Registry was utilized to identify all Finnish adolescents born between 1 January 1999 and 31 December 2000 (N = 10,476) who resided in Helsinki, and whose language was registered as Finnish (72% of the total sample, N = 7539; N = 3789 born in 1999 and N = 3750 born in 2000; 50% women). Of these invited, 1411 adolescents (19%) participated by responding to the online survey, and 1374 (18%) provided valid responses (66% women, *p* < 0.0001). Of these, we invited N = 552 with an emphasis on very late sleep rhythms with a self-reported bedtime after 1 a.m. at least 3 times a week (N = 364) and regular sleepers (N = 188). They were invited to participate in the next study phase, including a fixed period for the actigraphy (10 days) and circadian temperature (3 days) measurements; N = 353 (64%) agreed to participate, and N = 281 provided valid, complete circadian temperature data (N = 318 for sleep actigraphy) for the current study (70% women; mean (M) age = 17.4 years, Standard Deviation (SD) = 0.7; N_DSP_ = 144 (53%); N_regular sleep_ 121 (47%)). The present sample did not differ from the initial agreed cohort members regarding sex (*p* = 0.11) or age (*p* = 0.95). The measurement period of the study was conducted between November 2016 and December 2017. Informed consent was obtained from all participants. All procedures followed were in accordance with the Helsinki Declaration and its later amendments. Ethical permission was obtained from The Hospital District of Helsinki and Uusimaa Ethics Committee for gynaecology and obstetrics, pediatrics and psychiatry (Decision number 50/13/03/03/2016). The study is registered under Clinical Trials (ID: 1287174).

### Circadian temperature

Thermochron iButtons (DS1922 L, Maxim Integrated, San Jose, CA, USA) are small, light, stainless steel data loggers with thermometers. They contain digital thermistor sensors, which measure temperature with 0.0625 °C resolution and have the accuracy of ± 0.5 °C from − 10 to + 65 °C. They include memory for storing data on the temperature and time recordings, and can be initialized to the desired logging frequency.

In the current study, we selected the measurement rate to be one per minute, and the participants were instructed to wear the device for three days. The iButton was attached onto the wrist approximately upon the radial artery using adhesive medical tape. Participants were instructed how to re-attach the iButton if it was removed from the wrist, and advised to write down all times when this was done. The data were read with the USB Port Adapter as connected to the PC 1-Wire Connectivity Reader and extracted with the OneWireViewer software (Maxim Integrated, San Jose, CA, USA).

### Sleep actigraphy

The sleep variables were derived as mean measures over a 10-day logging period (M = 8 nights, SD = 1.6) in order to detect typical sleep. Participants were instructed to follow their normal sleeping patterns over the measurement period, and to report any atypical events or illnesses. The participating adolescents’ sleep was measured using actigraphy (GeneActiv Original, Kimbolton, UK).

The actigraphy data were cleaned from artefacts as described previously^38^. Sleep duration was calculated as the assumed total sleep time (i.e. the amount of time between sleep onset and wake-up time). Sleep quality was estimated by wake after sleep onset (WASO), which refers to the amount of minutes of wakefulness after falling asleep, and by sleep onset latency, which refers to the time between going to bed and falling asleep. Sleep timing was assessed by sleep onset and sleep offset times, as well as by calculating the midpoint of the sleep period. As measures of sleep regularity, we calculated the individual standard deviation of sleep onset and sleep midpoint over the measurement period.

### Circadian preference and delayed sleep phase

We used accelerometer data to gain an objective measure of subclinical DSP. We calculated a binary classification of concurrent DSP tendency based on going to sleep after 1 a.m. at least three times per week, as measured with actigraphy.

We assessed the behavioural circadian preference of each participant by using a short version of the original Morningness Eveningness Questionnaire (MEQ^39^), including six items (4, 7, 9, 15, 17 and 19) which yielded a sum score^40^. Higher scores indicate a more morning-oriented circadian preference. The six items had a Cronbach’s alpha of 0.70 in our data, which is slightly lower than the 0.80 reported previously in an adult population.

### Statistical analyses

The original circadian temperature recordings started between 3 p.m. to 10 p.m. and the starting time was harmonized such that all records were set to start from 12 p.m. to minimize the amount of missing data points and to remove the observed fluctuation in the temperature in the first hours of measurement. Missing values were imputed using regularized iterative principal components algorithm using the missMDA R-package^41^. Individual time series for each individual are presented in the Supplement Figures (S1).

**Figure 5:**
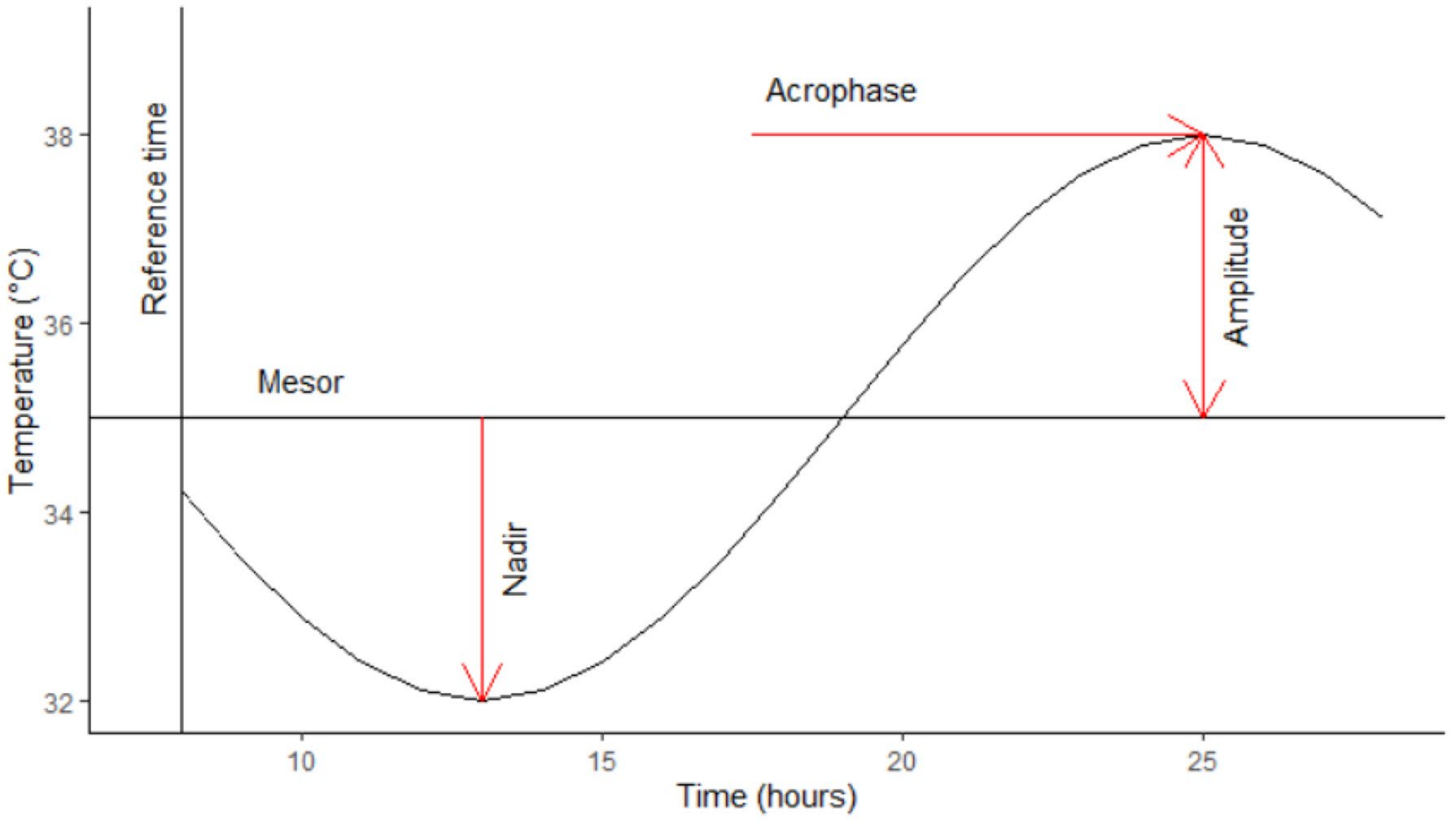
Interpretation of Cosinor parameters Mesor (rhythm-adjusted mean), Amplitude (half the extent of predictable variation within a cycle), Acrophase (the time of overall maximum values) and Nadir (the time of overall minimum values). Figure produced using R Core Team (2021). R: A language and environment for statistical computing. R Foundation for Statistical Computing, Vienna, Austria. URL https://www.R-project.org/.

Temperature measures were smoothed using loess (locally estimated scatterplot smoothing)^42^ using the span of 0.3. Cosinor model^43^ was fitted separately for each individual time series. The regression model for a single component cosinor model is written as:

$$ Y\left( t \right) = M + A\cos \left( {2\pi t/\tau + \phi } \right) + e\left( t \right) $$

where Mesor (M) is a rhythm-adjusted mean, amplitude (A) is a measure of half the extent of predictable variation within a cycle, acrophase (ϕ) is a measure of the time of overall maximum values recurring in each cycle, period (τ) is a duration of one cycle, and e(t) is the error term Interpretation of cosinor parameters is presented in Fig. 5. Nadir (bathyphase), which is a measure of overall minimum values recurring in each cycle, was estimated based on the cosinor model. The best fitting period of the cosinor model was estimated using iterative cosinor fitting^44^ and the acrophase was corrected using the method proposed by Bingham et al*.*^45^.

Sensitivity analysis was performed to assess how selection of time series smoothing method affects the results of cosinor analysis by repeating the time series smoothing using the Generalized additive model (GAM)^46^. Based on sensitivity analysis 32 observations were removed from subsequent analysis due to a large discrepancy between estimated cosinor parameters. After removal of those observations which were highly sensitive to the smoothing method, the final sample size was 281.

Smoothed Individual temperature time series were clustered using Partitioning Around Medoids (PAM) algorithm^47^, and a Discrete Fourier transformation was used as the dimension reduction method. Stability of clustering solution and number of clusters was further evaluated by comparing solutions to the results of machine learning algorithm t-distributed stochastic neighbor embedding (t-SNE)^48^ and network analysis based on Euclidean distances between Fourier parameters of individual time series.

The relationship between estimated temperature clusters and sleep outcomes was examined using linear and logistic regression analysis. To minimize the possibility of Type 1 error, confidence intervals and p-values were estimated using non-parametric bootstrapping with 500 bootstrapped resamples. Additionally, false discovery rate correction (FDR)^49^ was applied to all the results, taking into account all analysis. Pairwise group differences were assessed by comparing bootstrapped confidence intervals and post hoc analysis. All analysis was done using R-software version 4.02 (R Core Team, 2020).

## Supplementary Information

Supplementary Figure S1.

## Data Availability

The data is available at request from anukatriina.pesonen@helsinki.fi.
