# Supplementary material for: Data-driven modelling approach to circadian temperature rhythm profiles in free-living conditions
Source: Sci Rep. 2021 Jul 22;11:15029. doi: 10.1038/s41598-021-94522-9 (PMC8298484; doi:10.1038/s41598-021-94522-9)
Supplement: Supplementary file 1 — Supplementary Figure S1. [file 41598_2021_94522_MOESM1_ESM.pdf]

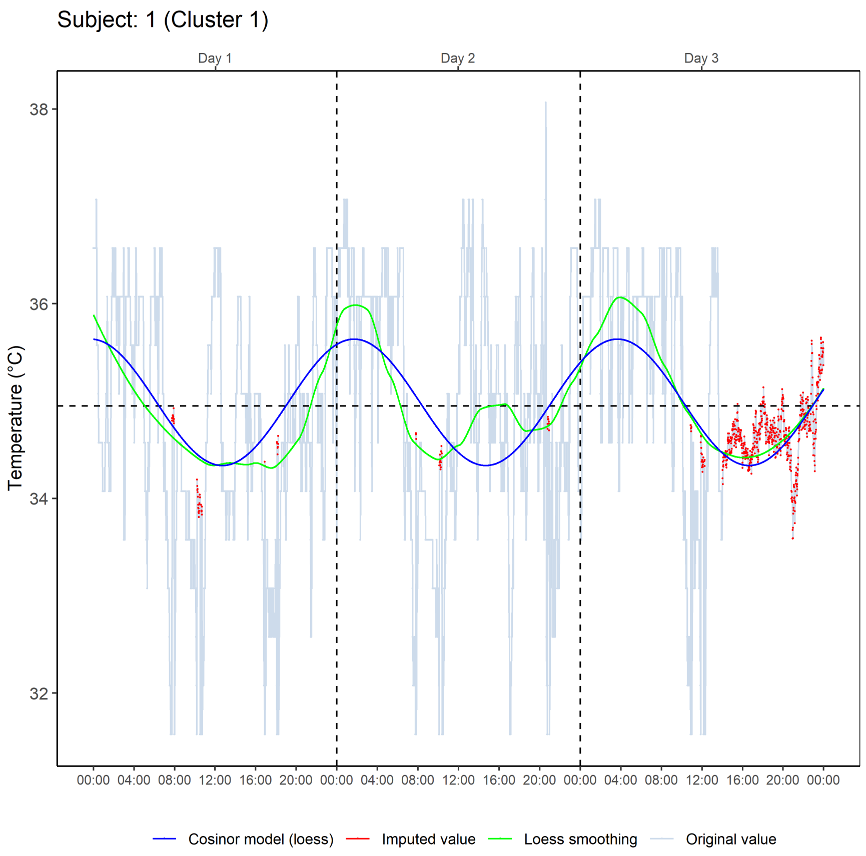

Supplement Figure S1: Individual observations, loess smoothing and predicted values of cosinor model based on the smoothed data. Horizontal dotted line equals average observed temperature over three days. Vertical lines indicate change of calender day. Red dots indicate imputed missing values which are imputed using the regularised iterative PCA algorithm (Josse and Husson, 2013). Use control buttons to review all figures in the animation. Cluster membership is mentioned in the title and ”Not included” indicates that subject is not included in the final analysis due to a missing values in sleep outcome variables or is rejected based on sensitivity analysis.
